# Further Evidence That People Rely on Egocentric Information to Guide a Cursor to a Visible Target

**DOI:** 10.1177/03010066211048758

**Published:** 2021-10-07

**Authors:** Emily M. Crowe, Martin Bossard, Harun Karimpur, Simon K. Rushton, Katja Fiehler, Eli Brenner

**Affiliations:** Department of Human Movement Sciences, Institute of Brain and Behavior Amsterdam, Amsterdam Movement Sciences, 1190 Vrije Universiteit Amsterdam, Amsterdam, The Netherlands; School of Psychology, 2112 Cardiff University, Cardiff, UK; Experimental Psychology, 9175 Justus Liebig University Giessen, Giessen, Germany; Center for Mind, Brain and Behavior (CMBB), University of Marburg and Justus Liebig University Giessen, Giessen, Germany; School of Psychology, 2112 Cardiff University, Cardiff, UK; Experimental Psychology, 9175 Justus Liebig University Giessen, Giessen, Germany; Center for Mind, Brain and Behavior (CMBB), University of Marburg and Justus Liebig University Giessen, Giessen, Germany; Department of Human Movement Sciences, Institute of Brain and Behavior Amsterdam, Amsterdam Movement Sciences, 1190 Vrije Universiteit Amsterdam, Amsterdam, The Netherlands

**Keywords:** pointing/hitting, perception/action, frames of reference, perturbation, allocentric

## Abstract

Everyday movements are guided by objects’ positions relative to other items in the scene (allocentric information) as well as by objects’ positions relative to oneself (egocentric information). Allocentric information can guide movements to the remembered positions of hidden objects, but is it also used when the object remains visible? To stimulate the use of allocentric information, the *position* of the participant’s finger controlled the *velocity* of a cursor that they used to intercept moving targets, so there was no one-to-one mapping between egocentric positions of the hand and cursor. We evaluated whether participants relied on allocentric information by shifting all task-relevant items simultaneously leaving their allocentric relationships unchanged. If participants rely on allocentric information they should not respond to this perturbation. However, they did. They responded in accordance with their responses to each item shifting independently, supporting the idea that fast guidance of ongoing movements primarily relies on egocentric information.

Many everyday tasks involve moving one’s fingers to a target. Such movements can rely on both egocentric and allocentric visual information (Colby, 1998). The weight given to allocentric information is modulated by various factors including cue reliability, landmark stability (Byrne & Crawford, 2010) and context (Klinghammer et al., 2015, 2017). However, evidence for the use of allocentric visual information comes from studies where the target of one’s movement is hidden. To investigate whether movements can also be guided by allocentric information when the target remains visible, we identified a task where relying on allocentric information might be particularly beneficial. Using a cursor rather than one’s hand to intercept a moving target should make relying on egocentric information more complicated, because the hand and cursor are located in different parts of space and move in different directions and by different amounts. However, this is not enough to make participants rely on allocentric information (Crowe et al., 2021). Do participants use allocentric information when the *velocity* of the cursor depends on the *position* of the participant’s finger? This complicated mapping should encourage participants to rely on allocentric information.

Twelve participants tried to guide a green cursor to intercept a black target that moved rightward at 30 cm/s across a large grey screen with randomly distributed black dots as the background (Figure 1a). The speed and direction of the cursor’s motion was determined by the position of the participant’s finger on the surface of a table in front of the screen: for each cm of finger displacement the cursor’s velocity changed by 6 mm/s in the corresponding direction. The finger’s position was recorded at 500 Hz with an Optotrak 3020 (Northern Digital). From 300 ms after the target appeared, the target, cursor and background could be subjected to additional lateral motion at 20 cm/s for 100 ms. There were four conditions: one in which all three items were subjected to the same additional motion and three in which only one item was subjected to such motion. If participants rely on allocentric information they should not respond when all three items are subjected to the same additional motion such that their relative positions do not change. If they rely on egocentric information the response to all three items being subjected to the same additional motion should be the sum of the responses to each item being subjected to such motion independently. For each condition, the additional motion was leftward on 50 trials and rightward on 50 trials, giving a total of 400 trials per participant. The trials were interleaved and participants received feedback about whether they hit the target. For each participant we determined the *response* to each kind of perturbation by subtracting the average lateral position of the finger (corresponding with the average lateral velocity of the cursor) for leftward perturbations from that for rightward perturbations.

**Figure 1.**
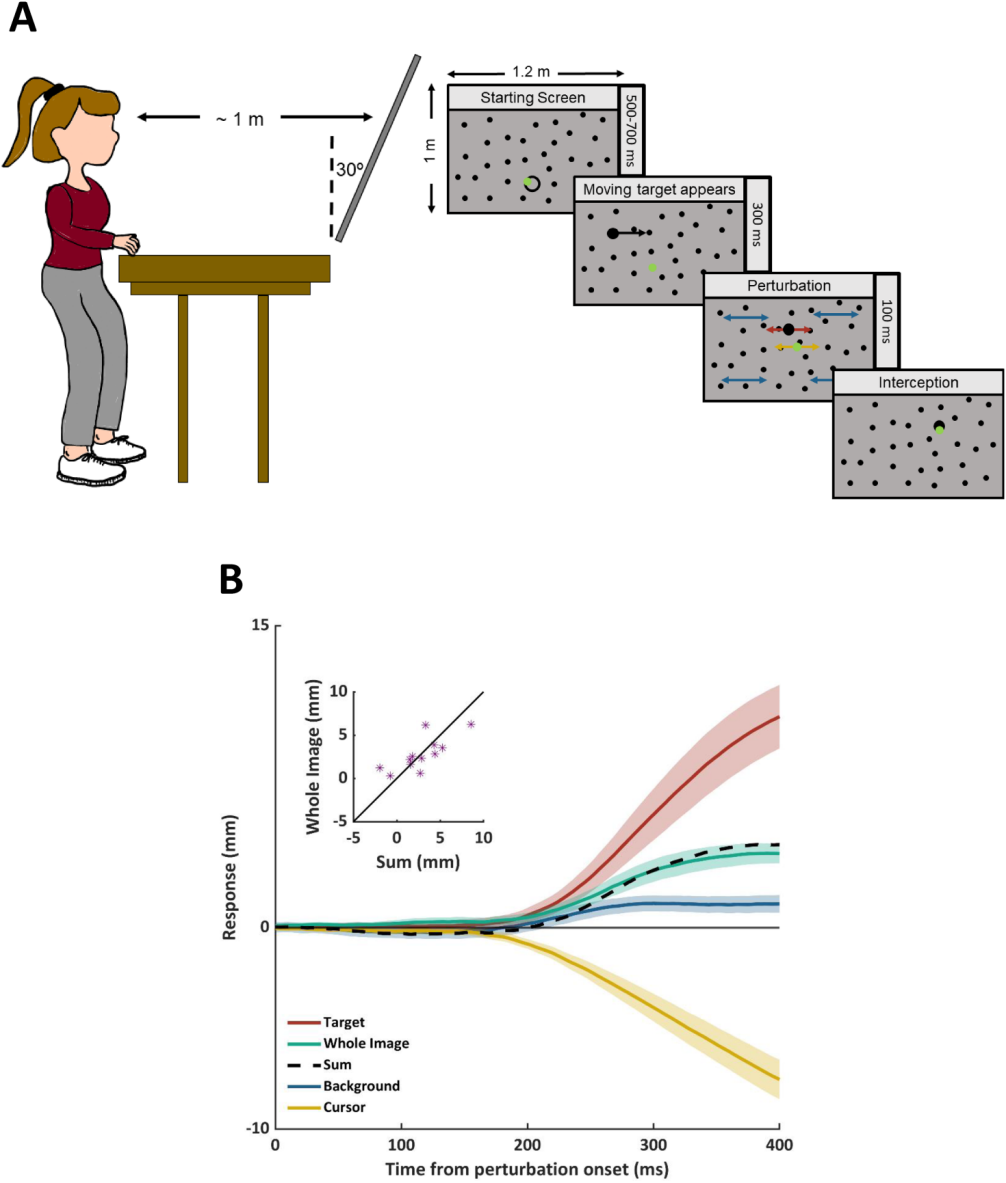
(a) Schematic representation of the set-up (left panel) and task (right panel). (b) Coloured curves show the average responses in each condition. A positive response is in the direction of the perturbation and a negative response is in the direction opposite the perturbation. Shaded regions show the standard error across participants. The black curve shows the sum of the average responses to the separate background, target, and cursor perturbations. The inset compares this sum with the response to the whole image moving for individual participants (300 ms after perturbation onset).

We reasoned that introducing a complicated mapping between finger and cursor movements would make it more difficult to use egocentric information to guide one’s movements thus encouraging participants to rely on allocentric information. If participants used allocentric information they should not have responded to the whole image shifting because the allocentric relations were unchanged such that no adjustment to the ongoing movement was required. However, participants did respond to this perturbation. In line with Crowe et al. (2021), the *sum* of the responses to the three individual perturbations closely matched the response to the whole image shifting (Figure 1b). When only one of the three items was perturbed participants responded appropriately: in the direction of *target* perturbations and in the opposite direction to *cursor* perturbations. They also responded to *background* perturbations, as has previously been observed.

Why does perturbing the background influence performance if only egocentric positions are relevant, and the task is to bring the cursor to the target? There may be a transient shift in the egocentric position of the anticipated movement endpoint, mimicking the shift that would occur if the changing egocentric position of the background were due to self-motion. Considering such shifts might be particularly important when the target is occluded or hidden due to the experimental design. This explains why only motion of certain items (Klinghammer et al., 2017) or items in certain regions (Brenner & Smeets, 2015) is relevant. The finding that participants respond to all items moving together, and even do so very similarly to the sum of how they respond to each item moving independently, reinforces the idea that ongoing movements to visible targets are primarily guided by egocentric information even when there is no anatomical link between one’s actions and their consequences (Crowe et al., 2021). This has implications for the optimisation of human-device interaction systems, because it can help understand why certain links between the user and the system they are controlling are more intuitive than others.
